# Characteristics of Donor-Specific Antibodies Associated With Antibody-Mediated Rejection in Lung Transplantation

**DOI:** 10.3389/fmed.2017.00155

**Published:** 2017-10-11

**Authors:** Antoine Roux, Ines Bendib Le Lan, Sonia Holifanjaniaina, Kimberly A. Thomas, Clément Picard, Dominique Grenet, Sandra De Miranda, Benoit Douvry, Laurence Beaumont-Azuar, Edouard Sage, Jérôme Devaquet, Elise Cuquemelle, Morgan Le Guen, Caroline Suberbielle, Chantal Gautreau, Marc Stern, Maura Rossetti, Abdul Monem Hamid, Francois Parquin

**Affiliations:** ^1^Pneumology, Adult CF Center, Lung Transplantation Department, Foch Hospital, Suresnes, France; ^2^Department of Pathology and Laboratory Medicine, University of California, Los Angeles, Los Angeles, CA, United States; ^3^Université Versailles Saint-Quentin-en-Yvelines, Montigny le Bretonneux, France; ^4^Pathology Department, Foch Hospital, Suresnes, France; ^5^Thoracic Surgery Department, Foch Hospital, Suresnes, France; ^6^Intensive Care Unit, Foch Hospital, Suresnes, France; ^7^Thoracic Intensive Care Unit, Foch Hospital, Suresnes, France; ^8^Anesthesiology Department, Foch Hospital, Suresnes, France; ^9^Laboratoire Régional d’Histocompatibilité, Saint-Louis Hospital, Assistance Publique-Hôpitaux de Paris, Paris, France

**Keywords:** HLA, donor-specific antibodies, lung transplant, clinical outcome, antibody mediated rejection

## Abstract

Although donor-specific anti-human leukocyte antigen (HLA) antibodies (DSAs) are frequently found in recipients after lung transplantation (LT), the characteristics of DSA which influence antibody-mediated rejection (AMR) in LT are not fully defined. We retrospectively analyzed 206 consecutive LT patients of our center (2010–2013). DSAs were detected by using luminex single antigen beads assay and mean fluorescence intensity was assessed. Within the study population, 105 patients had positive DSA. Patients with and without AMR (AMR^Pos^, *n* = 22, and AMR^Neg^, *n* = 83, respectively) were compared. AMR^Pos^ patients had significantly greater frequencies of anti-HLA DQ DSA (DQ DSA) than AMR^Neg^ patients (95 vs 58%, respectively, *p* < 0.0001). Compared to AMR^Neg^ patients, AMR^Pos^ patients had higher DQ DSA sum MFI [7,332 (2,067–10,213) vs 681 (0–1,887), *p* < 0.0001]. DQ DSA when associated with AMR, had more frequent graft loss and chronic lung allograft dysfunction (CLAD). These data suggest (i) that DSA characteristics clearly differ between AMR^Pos^ and AMR^Neg^ patients and (ii) the deleterious impact of DQ DSA on clinical outcome.

## Introduction

The role of donor-specific anti-human leukocyte antigen (HLA) antibody (DSA) in graft failure *via* antibody-mediated rejection (AMR) and sub-clinical chronic AMR has been widely established in kidney transplantation (1–3) and heart transplantation (4) (KT and HT, respectively). Our group (5) and others (6, 7) demonstrated in the context of lung transplantation (LT) that AMR was associated with chronic lung allograft dysfunction (CLAD) and poor graft survival. Previous studies concerning the prognostic value of DSA, despite the significant contribution of knowledge regarding DSA in solid organ transplantation (SOT), provide only limited characterization of DSA in the setting of LT. As a matter of fact, solid phase assay as luminex single antigen beads (SAB) assay cannot be used for quantization of the DSA strength but allow determination of appropriate mean fluorescence intensity (MFI) threshold for DSA identification or their impact on clinical status.

Two retrospective analyses of KT cohorts (8, 9) have shown that presence of pre-transplant (preTx) DSA and MFI of the immunodominant DSA (understood as the DSA with the highest MFI for a given patient) were associated with graft loss. These publications did not further describe DSA either post-transplant (postTx) or at the moment of AMR. Comparison of DSA MFI from SAB tests and from a complement binding test suggested that the MFI of immunodominant DSA or the sum MFI of all DSA may be as efficient as the complement binding test for AMR prediction and graft failure (10). More recently, Tikkanen et al. showed that particularly HLA DQ mismatch and subsequent DQ DSA were associated with CLAD, yet the results did not show if the MFI was also associated with poor graft outcome (11). Moreover, these studies did not integrate potential AMR occurrence associated with DQ DSA for the analysis of graft outcome.

In this study, we propose an extended analysis of DSA characteristics in our cohort previously described for AMR impact on graft prognosis (5). We took advantage of our extensive DSA monitoring strategy and prospective assessment of AMR diagnosis to analyze DSA characteristics according to AMR status and thereby evaluate their diagnostic performance and evaluate the clinical outcomes associated with DQ DSA.

## Materials and Methods

### Patient Population, DSA Monitoring Strategy, and HLA Testing

All consecutive patients of the lung transplant cohort in Foch Hospital from January 2010 to December 2013 were eligible. Patients were routinely tested for HLA antibody (HLA-Ab) postTx at days 1, 7, 21, and 30; at months 2, 3, 4, 6, 9, and 12; and then every 6 months thereafter. From January 2010 to December 2012, patients were tested by first tested by LabScreen Mixed^®^ (LSM, One Lambda) at these scheduled time point. At least once in the first 3 months and at month 12, and if positivity of Labscreen Mixed or graft failure, serum were further tested with LabScreen Single Antigen^®^ (LSA, One Lambda, Canoga Park, CA, USA). After December 2012, patients were systematically tested by LSA at each time point. In our analysis, negative results of either LSM or LSA were reported as negative for DSA. Patient’s HLA typing was done using standard molecular biology techniques (SSO, One Lambda^®^) and then reported as serological equivalents in clinical reports. The One Lambda kits were used according to manufacturer’s recommendations. Deceased donor’s HLA typing was performed by serological typing and/or molecular biology (PCR-SSP) according to the European Federation of Immunogenetics rules.

Donor-specific antibody positivity was defined if the beads loaded with donor HLA antigen specificity had MFI >500. Specificity is assigned considering the highest MFI bead when several beads express the same antigen.

### Analysis of DSA Characteristics

The immunodominant DSA was defined as the DSA with highest MFI in a given serum sample. DQ DSA specificities were reported for HLA-DQB only. The peak was defined as the time point with the highest sum MFI for AMR^Neg^ patients or the time point of AMR diagnosis for AMR^Pos^ patients. At the peak, we compared the number of DSA specificities, the MFI of the immunodominant, Class I, Class II, DQ DSA, preformed, and *de novo* DSA, and the sum MFI between AMR^Pos^ and AMR^Neg^ patients.

### AMR Categorization

Antibody-mediated rejection categorization was established prospectively, as described in our previous publication (5), by a multidisciplinary physician and pathologist team using the assessments listed below. All AMR cases met criteria of ISHLT consensus for definite or probable DSA positive AMR (12). Briefly, patients with AMR (AMR^Pos^) were defined by three criteria: (i) the presence of HLA DSA (DSA^Pos^: DSA MFI >1,000, or MFI = 500–1,000 with more than two specificities and/or detected more than once), (ii) biopsy patterns relative to AMR [including positive C4d staining and/or histological pattern compatible with AMR (i.e., neutrophil capillaritis, or acute lung injury with or without organizing pneumonia)], and (iii) graft failure (−20% decrease in forced expiratory volume in 1 second or hematosis degradation requiring introduction/increase of oxygenotherapy or mechanical ventilation). DSA^Pos^ patients without graft failure and biopsy patterns indicative of AMR were defined as AMR^Neg^ (Table 1).

**Table 1:** Criteria for antibody-mediated rejection-donor-specific antibody status categorization.

Antibody-mediated rejection (AMR) patients (DSA^pos^AMR^pos^)		Non-AMR patients		
		AMR^neg^ (DSA^pos^AMR^neg^)	DSA^Lim^	DSA^neg^
Donor-specific antibody (DSA) positivity [DSA mean fluorescence intensity (MFI) > 1,000, or MFI = 500–1,000 with more than two specificities, and/or detected more than once]		DSA positivity (DSA MFI > 1,000, or MFI = 500–1,000 with more than two specificities, and/or detected more than once)	DSA detected only once and having only one specificity with an MFI = 500–1,000	All single antigen tests with DSA MFI < 500

**AMR C4d^pos^**	**AMR C4d^neg^**	**No AMR diagnosis through entire follow up**		

Clinical dysfunction and DSA positivity and C4d positive staining with or without histological patterns suggestive of AMR	Clinical dysfunction and DSA positivity and negative C4d staining with histological patterns suggestive of AMR: neutrophil capillaritis^a^; acute lung injury^a^			

*^a^At the exclusion of other diagnoses (ischemia–reperfusion, infection, aspiration, and drug toxicity)*.

### Clinical Outcome

For clinical outcome analysis we used (i) graft survival conditioned by 3-month survival, defined as graft survival among patients alive at month 3 allowing enough followup to consider the absence of DSA or DQ DSA as meaningful (ii) freedom from CLAD (chronic lung allograft dysfunction) as defined previously (13) within patients alive at 6 months and excluding patient with bronchial issue as previously reported (5).

### Ethics

This observational study was approved by the research protocol evaluation committee of the Institutional Review Board of the French Learned Society for Respiratory Medicine—Société de Pneumologie de Langue Française. Every patient in this study was enrolled in a prospective non-therapeutic interventional research protocol [either COLT (NCT00980967, ID-RCB:2009-A00036-51) or RhumTP (NCT01791166, ID-RCB:2010-A01174-35)]. Upon enrollment, all patients signed consent for research use of their clinical data.

### Statistical Analysis

Categorical variables were expressed as number and percentage and compared by Chi- square test or Fisher’s exact test as appropriate. Quantitative variables were expressed as median and interquartile 25-75 (IQR 25-75) or mean and 95% confidence interval (CI 95%) or SD and compared by Mann–Whitney or paired Wilcoxon signed-rank test for repeated measures. Area under the curve (AUC), specificity, and sensitivity of MFI for AMR diagnosis were determined using the ROC method. Survival analysis was calculated by log-rank test.

All analyses were performed using Prism^®^ v5.0 for Mac OS X (Graphpad Software, San Diego, CA, USA). A *p*-value below 0.05 was considered statistically significant.

## Results

### Study Population

**Figure 1:**
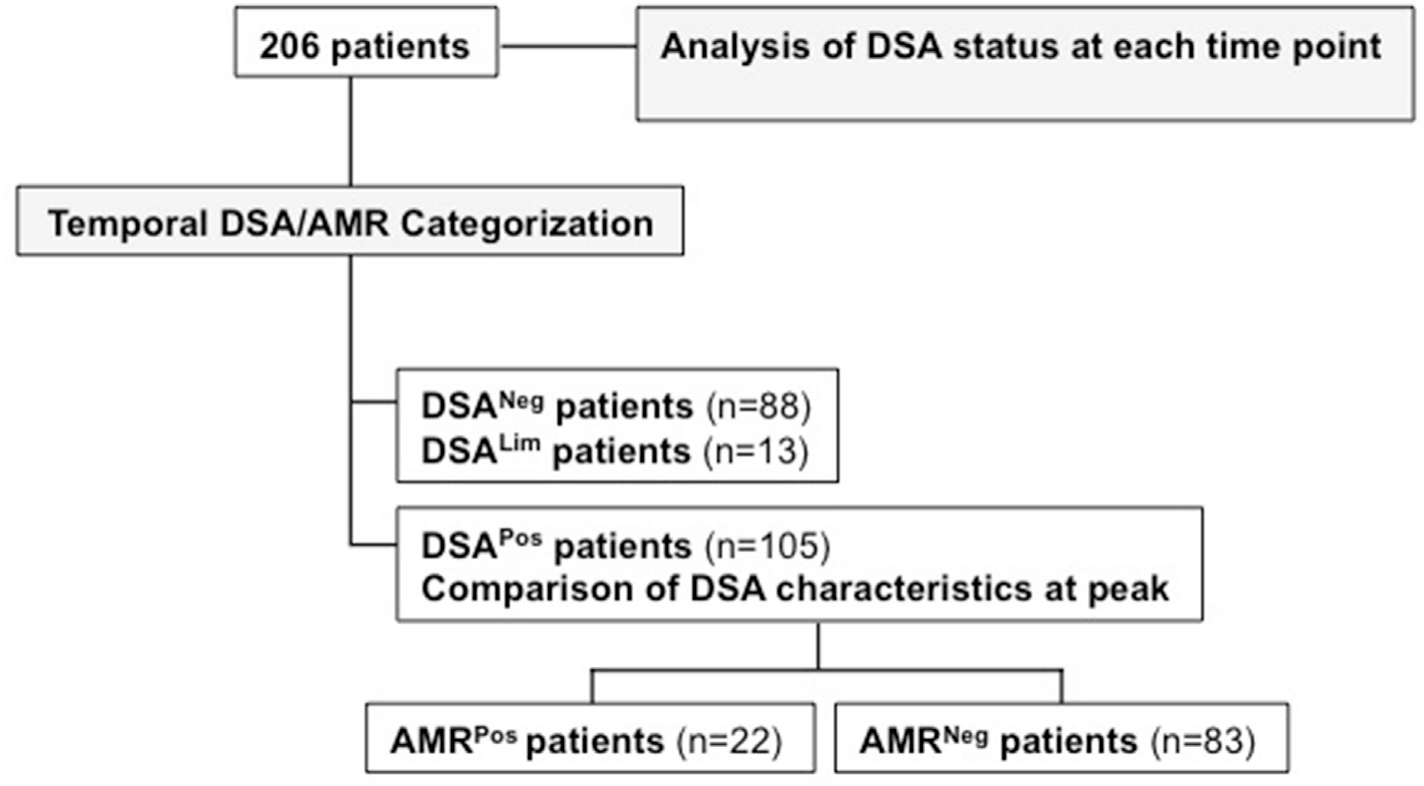
Flow chart and analysis summary.

Among 209 eligible patients, 206 were included in the analysis. Of these 206, 88 patients had no detectable DSA during the entire followup period (DSA^Neg^), 13 had one DSA once with an MFI 500–1,000 (DSA^Lim^), and 105 patients were DSA^Pos^. Within the DSA^Pos^ group, 22 patients were prospectively diagnosed with AMR (AMR^Pos^), while 83 had at least one positive DSA detection during the whole followup but no AMR diagnosis (AMR^Neg^) (Figure 1). AMR^Pos^ and AMR^Neg^ patients did not differ for clinical baseline characteristics except for the number of HLA mismatches, which was higher in AMR^Pos^ patients (Table 2). Interestingly, the frequency of patients having two DQ mismatches significantly differed between AMR^Pos^ (68%) and AMR^Neg^ patients (26.5%) but the frequency of presensitized patients did not differ between the two group (12 (54%) vs. 58 (69%), respectively).

**Table 2:** Baseline clinical characteristics.

	AMR^Pos^ patients	AMR^Neg^ patients	*p*
Recipient age, median (IQR 25-75)	35.45 (23.65–53.25)	36.30 (27.7–51.3)	0.78
Underlying disease (CF/PF/COPD/other), *n*	10/5/4/3	45/15/15/8	0.86
Recipient female, *n* (%)	11 (50)	44 (53)	0.45
LAS, median (IQR 25-75)	36.85 (33.2–42.1)	38 (35.1–43.7)	0.44
HLA mismatch, mean (SD)	6.9 (0.7)	5.9 (1.2)	<0.001
PreLT DSA, *n* (%)	12 (54)	58 (69)	0.2
Induction therapy, *n* (%)	12 (54)	52 (62.3)	0.62

*Categorical variables were compared by Fisher’s exact test. Quantitative variables were compared by Mann–Whitney test. HLA mismatching was defined as the number of donor HLA antigens among loci A, B, DRB1, DQB1 differing from recipient antigens (4 loci × 2 alleles each = maximum of 8 mismatches)*.

*CF, cystic fibrosis; COPD, chronic obstructive pulmonary disease; LAS, lung allocation score; PF, pulmonary fibrosis*.

### Immunodominance As an Indicator of AMR

**Figure 2:**
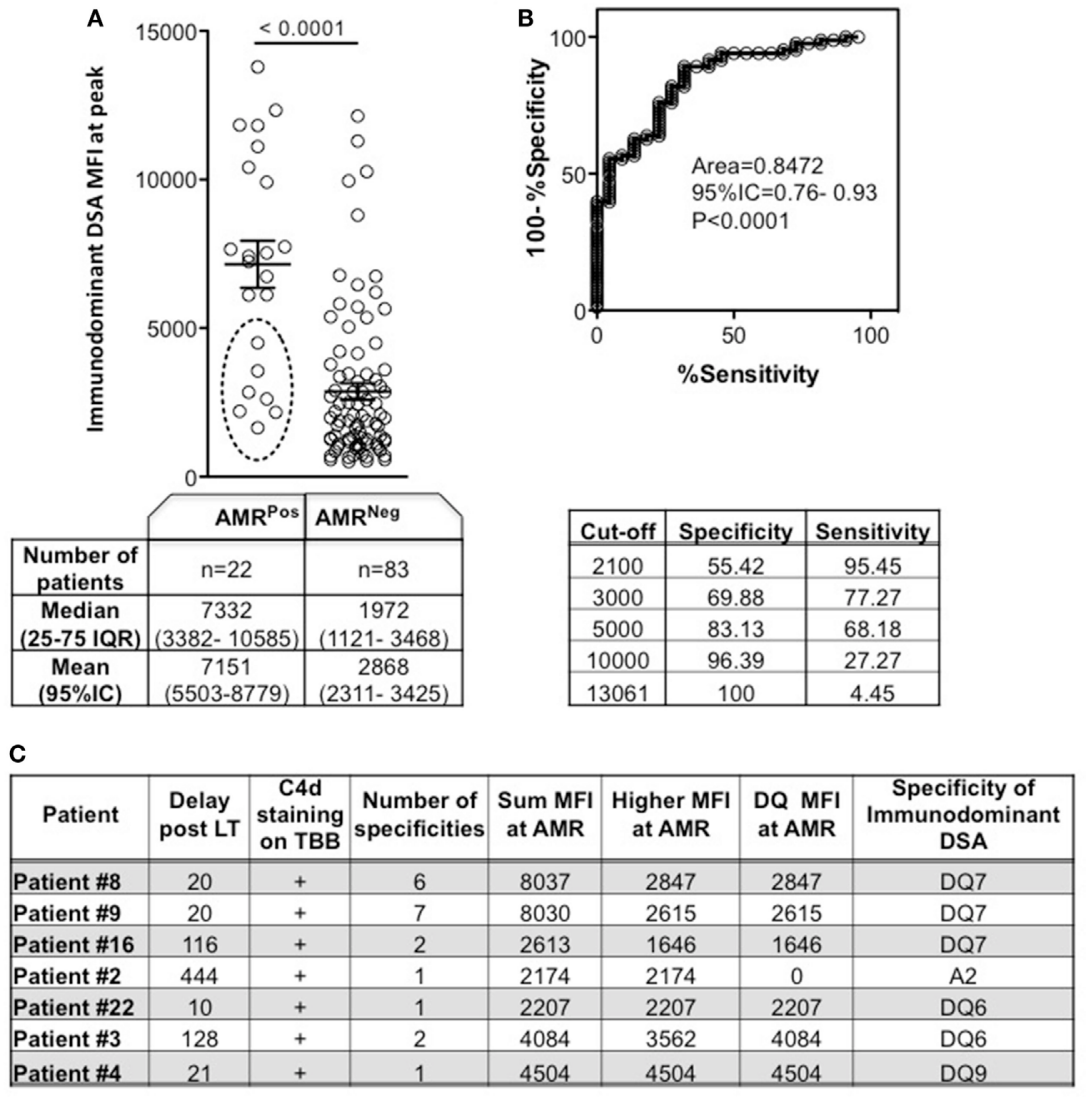
Mean fluorescence intensity (MFI) of immunodominant donor-specific antibody (DSA) at peak according to antibody-mediated rejection (AMR) status. Peak was defined as the time point of DSA with the highest sum MFI for AMR^Neg^ (DSA^Pos^AMR^Neg^) patients, or as the time of AMR diagnosis for AMR^Pos^ patients. **(A)** Each patient’s immunodominant DSA is a circle. Circles inside broken line ellipse (*n* = 7) represent patients with AMR despite an immunodominant DSA MFI < 5,000, whose characteristics are detailed in panel **(C)** (Mann–Whitney test). **(B)** ROC curve for use of immunodominant DSA MFI as a predictor of AMR diagnosis, and the specificity/sensitivity according to different MFI cut-offs. **(C)** Characteristics of the 7 AMR^Pos^ patients with immunodominant DSA MFI < 5,000. Significance values are denoted above lines over compared groups.

The immunodominant DSA MFI at the peak time point was significantly higher for AMR^Pos^ than for AMR^Neg^ patients (Figure 2A). Given the significant difference and high deviation for either median or mean values between AMR^Pos^ and AMR^Neg^, we evaluated the accuracy of using the immunodominant DSA MFI for AMR diagnosis. The AUC was 0.84, with an estimated sensitivity of 95.45% with a low cutoff (2,100) and specificity of 100% with an upper cutoff (13,061) (Figure 2B). The seven AMR^Pos^ patients with immunodominant MFI < 5,000 are more precisely described in Figure 2C: all patients had DSA associated with C4d + AMR; all except one had DQ dominant DSA; and 4 out of 7 had greater than two different specificities. Of note sera with low MFI from AMR^Pos^ patients were all retested with EDTA and showed similar MFI excluding prozone effect for those patients.

### Sum MFI As an Indicator of AMR

**Figure 3:**
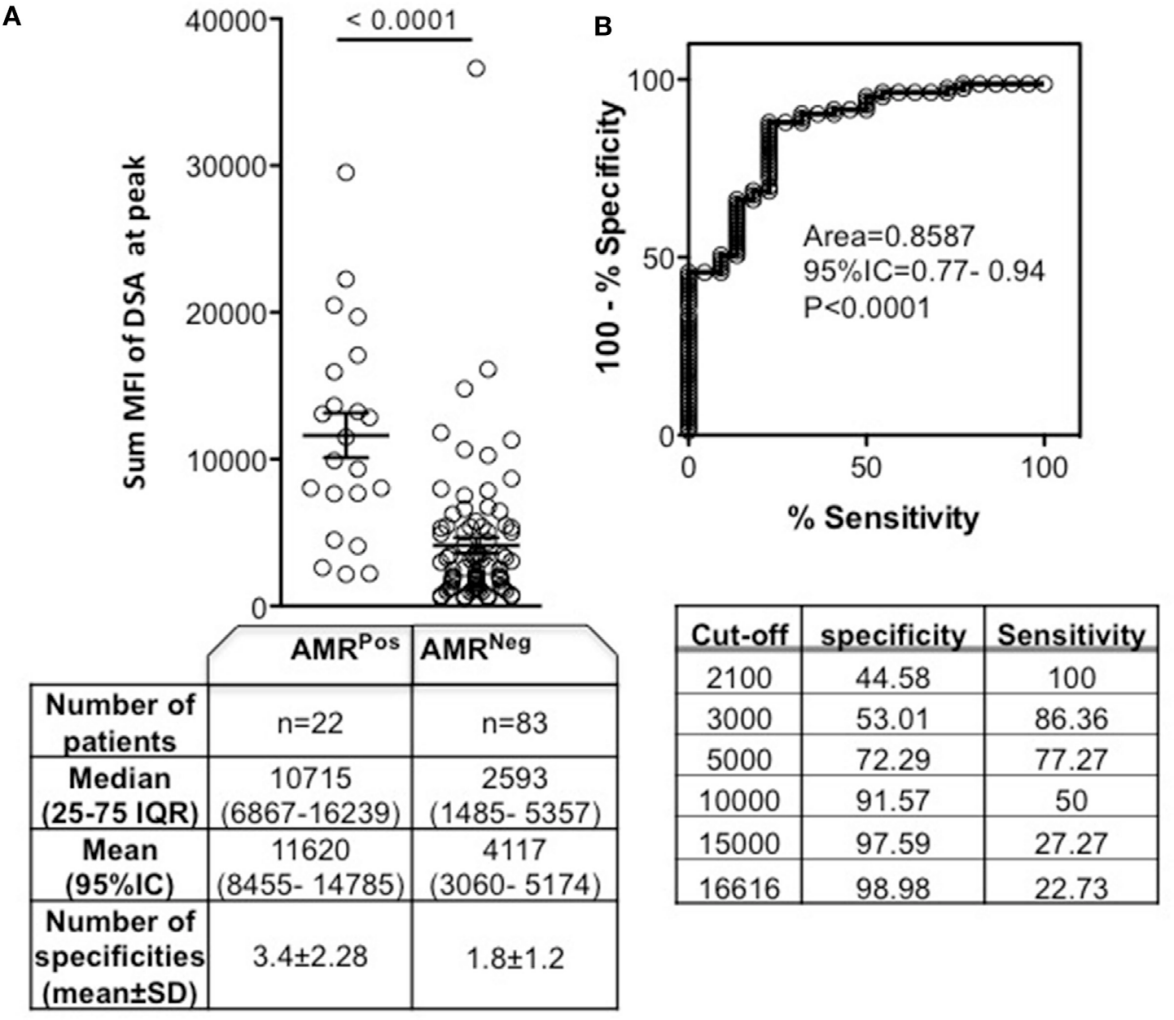
Sum mean fluorescence intensity (MFI) of donor specific antibody (DSA) at peak according to antibody-mediated rejection (AMR) status. Peak was defined as the time point of DSA with the highest sum MFI for AMR^Neg^ (DSA^Pos^AMR^Neg^) patients, or as the time of AMR diagnosis for AMR^Pos^ patients. **(A)** The sum MFI of each patient’s DSA is indicated by each circle. The table below lists the number of patients, median and mean sum MFI, and number of specificities for each patient group. **(B)** ROC curve for use of sum MFI as a predictor of AMR diagnosis and the specificity/sensitivity according to MFI different cutoffs. Significance values are denoted above lines over compared groups.

Despite the use of immunodominant DSA as an important indicator of AMR status, that particular DSA characteristic did not provide information regarding the number of DSA specificities. Compared to AMR^Neg^, AMR^Pos^ patients had significantly increased number of DSA specificities (3.4 ± 2.28 vs 1.8 ± 1.2, *p* = 0.0013) (Figure 3A). As this dimension has been shown to contribute to antibody pathogenicity (9), we then analyzed the sum MFI for each patient at the peak time point to account for the total number of specificities. The sum MFI of AMR^Pos^ patients was significantly higher than the sum MFI of AMR^Neg^ patients (Figure 3A). Similar to what was seen above with immunodominant MFI, sum MFI showed high diagnostic performance for AMR with 100% sensitivity at a lower cutoff (2,100) and more than 97% specificity at an upper cutoff (15,000) (Figure 3B). This assessment of sum MFI, regardless of DSA specificity or preformed/*de novo* nature, provides an interesting diagnostic performance for AMR diagnosis.

### DQ DSA As an Indicator of AMR and As a Determinant of Outcome

**Figure 4:**
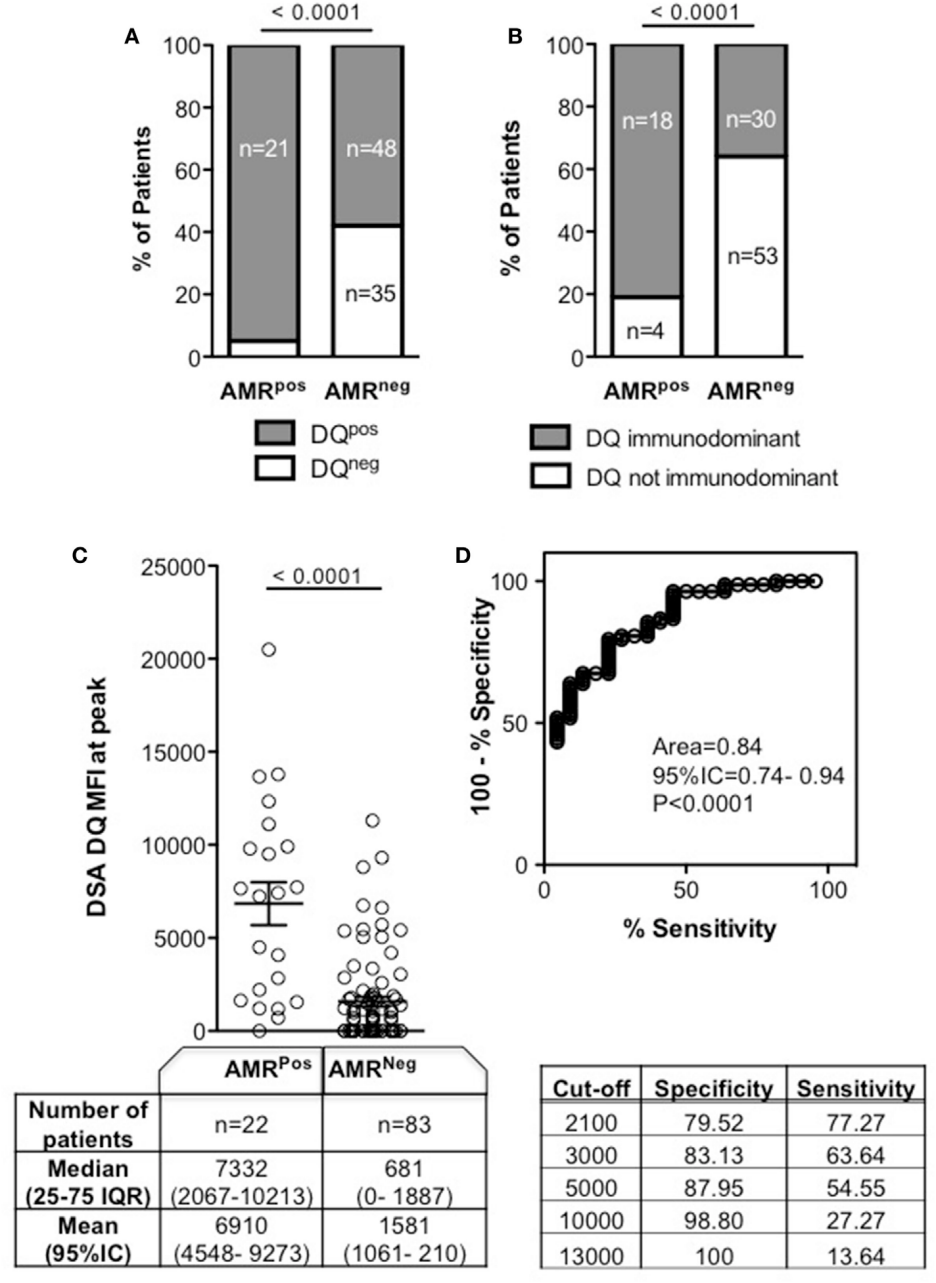
DQ mean fluorescence intensity (MFI) of donor specific antibody (DSA) at peak according to antibody-mediated rejection (AMR) status. Peak was defined as the time point of DSA with the highest sum MFI for AMR^Neg^ (DSA^Pos^AMR^Neg^) patients, or as the time of AMR diagnosis for AMR^Pos^ patients. **(A)** Frequency and number of patients with DQ DSA at the peak. **(B)** Frequency and number of patients with immunodominant DQ DSA at the peak. **(C)** Each patient’s DQ DSA MFI are represented as a circle. Comparison of number of patients, as well as median and mean DQ DSA MFI, for AMR^Pos^ and AMR^Neg^ patients is in the table below. **(D)** ROC curve for use of DQ DSA MFI as a predictor of AMR diagnosis and the specificity/sensitivity according to different MFI cutoffs. Groups were compared using Chi-square **(A,B)** and Mann–Whitney tests **(C)** and significance values are denoted above lines over compared groups.

As DQ specificity has been previously described (14) as a characteristic which correlates with AMR, we looked for an association of DQ DSA with AMR in our cohort. All AMR^Pos^ patients had DQ DSA except one (95%) who was matched with donor DQ, compared to only 58% of AMR^Neg^ patients (*p* < 0.0001) (Figure 4A). The immunodominant DSA was specific for DQ in 18/22 (81%) AMR^Pos^ patients, but only in 30/83 (36%) AMR^Neg^ patients (*p* < 0.0001, Figure 4B). For the four remaining AMR^Pos^ patients, the immunodominant specificities were A2, Cw05, and DR13 (twice). The MFI of DQ DSA was higher in AMR^Pos^ patients (Figure 4C) and the diagnostic performance of DQ DSA MFI for AMR was similar to those of both the immunodominant and the sum MFI (Figure 4D).

**Figure 5:**
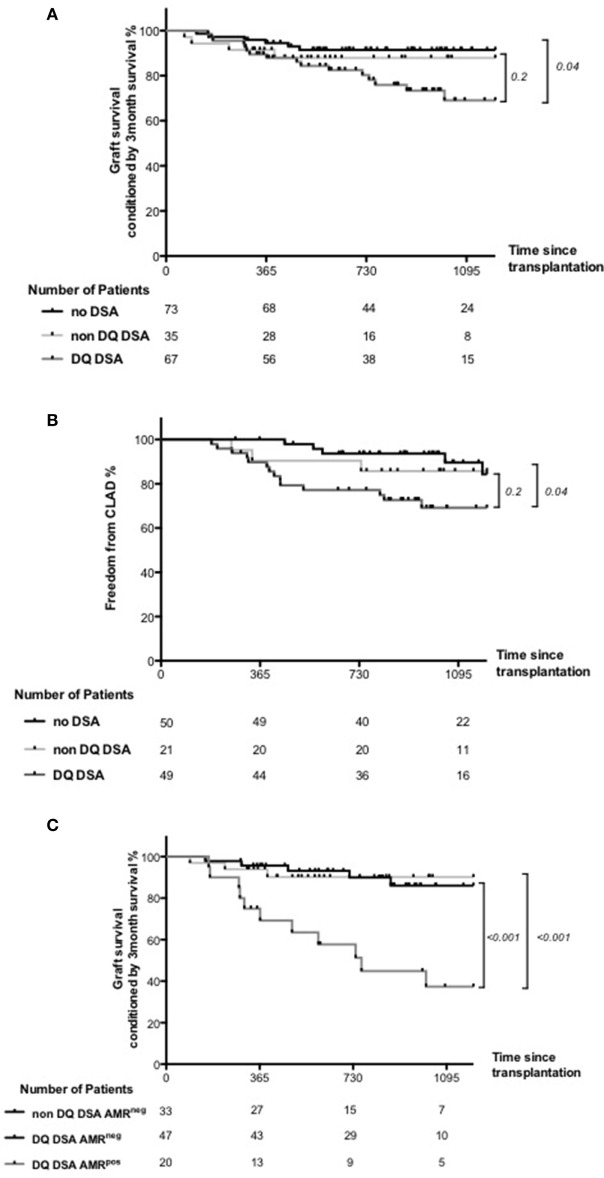
Clinical outcome associated with anti-HLA DQ donor-specific antibody (DQ DSA). Patients were categorized as DQ DSA if they had at least once DSA directed against one DQ antigen. Patients were categorized as non DQ DSA if they had DSA but never directed against DQ antigen. **(A)** Graft survival conditioned by 3-month survival compared between DSA negative, non-DQ DSA and DQ DSA patients. Global comparison was statistically significant, but non-DQ DSA and DQ DSA comparison did not reach significance. **(B)** CLAD occurrence between DSA negative, non-DQ DSA, and DQ DSA patients. Again, global comparison was statistically significant, but non-DQ DSA and DQ DSA comparison did not reach significance. **(C)** Graft survival conditioned by 3-month survival compared between non DQ DSA AMR^neg^, DQ DSA AMR^neg^, and DQ DSA AMR^pos^ patients. The only non-DQ DSA AMR^pos^ patient was exclude of the analysis. Both global and DQ DSA AMR^neg^ and DQ DSA AMR^pos^ comparison were highly significant.

Given the previously shown negative impact of DQ DSA on lung transplant outcome (11), we evaluate the association of DQ DSA with graft survival conditioned by the 3 months survival and CLAD occurrence. Compared to DSA negative patients and non-DQ DSA patients, DQ DSA patients had significantly worst graft survival conditioned by the 3 months survival and more frequent CLAD occurrence (Figures 5A,B). Of note, this comparison did not reach significance when comparing only non DQ DSA and DQ DSA patients.

Finally and more importantly, by splitting patients according to their DQ DSA and AMR status, the analysis showed a clearly worst outcome within DQ DSA AMR^pos^ patients than patients both DQ and non DQ AMR^Neg^ patients (Figure 5C).

## Discussion

Our extensive analysis provides a global overview of DSA frequency after LT and shows a multidimensional discrepancy of DSA characteristics between AMR^Pos^ and AMR^Neg^ LT patient populations. In our patient categorization, AMR^Pos^ patients match the new consensus criteria of definite (C4d positive DSA positive) or probable (C4d negative DSA positive) clinical AMR (12). Both immunodominant DSA MFI, sum MFI and DQ DSA MFI show high diagnostic performance for distinguishing between AMR^Pos^ and AMR^Neg^ patients. Moreover, DQ DSAs are associated with worse clinical outcomes through the occurrence of AMR.

Considering the cumulative DSA status of our cohort, 50% of patients were DSA^Pos^. These results are in line with previous reports (6, 15, 16).

We found AMR^Pos^ patients to have significantly higher DSA MFI (either immunodominant or sum MFI) than their AMR^Neg^ counterparts. The MFI values for AMR^Pos^ patients in our cohort were similar to those described by Lobo et al. (7), but lower than those values found in KT studies (9). The lower MFI values reported in LT may be partly due to the “sponge effect” related to the higher capillary surface in the lung. Of note, DSA adsorption in the graft was first described decades ago (17), but the real impact of this phenomenon on DSA underestimation remains unclear. Futures studies analyzing intragraft DSA may bring new information about the reality of sponge effect in LT (18). Alternatively, there may be an organ-specific susceptibility to DSA, due either to varying expression levels of complement inhibitory receptors and HLA molecules or differential responses to DSA ligation of HLA depending on the microvascular bed (19).

Sum MFI integrate the number of specificities for a given patient. Being aware that this summation only approximately represents the biological complexity of antibody–antigen interaction, in the case of multiple DSA, the sum MFI may be a more appropriate estimation of the DSA load than immunodominant DSA MFI alone.

In line with a previous publication (14), we also found that DQ DSAs were overrepresented in AMR^Pos^ patients. Not only, we demonstrate a specific DQ DSA effect on graft survival and CLAD, as Tikkanen did (11), but our results suggest also that these poor clinical outcomes were mainly associated with AMR occurrence.

Taken together, these results suggest (i) a specific association between DQ and AMR and (ii) that the negative impact of DQ DSA could be mainly driven by AMR occurrence. Consequently, whether DQ matching could prevent AMR occurrence warrants further investigation.

Importantly, AMR also occurred in the absence of DQ DSA indicating that DQ DSA is not a prerequisite for AMR. On the other hand, the fact that every DQ DSA does not necessarily lead to AMR emphasize our need for better characterization of pathogenic DSA. ROC evaluation suggests that the DSA MFI (sum MFI, immunodominant MFI, and DQ MFI) could help to identify AMR^Pos^ and AMR^Neg^ patients at the peak time point. To our knowledge, this is the first report of this kind.

However, the overlap between AMR^Pos^ and AMR^Neg^ patients was consistent, as 50% of the AMR^Pos^ patients and 40% of AMR^Neg^ patients had immunodominant MFI between 2,100 and 10,000, and 68% of the AMR^Pos^ patients and 47% of AMR^Neg^ patients had a sum MFI between 2,100 and 10,000.

Besides the large overlap limitating the diagnosis value of MFI, either immunodominant or sum MFI show a very high sensitivity for AMR when considering a 10,000 cutoff.

In general, putative explanations for AMR^Pos^ patients with a “low” MFI include the prozone effect (20), the aforementioned sponge effect, and concurrent undetected non-HLA DSA.

Altogether, these findings should be interpreted with caution and serve as an advocate for interpreting DSA results in the context of graft clinical status. In addition, single antigen tests performed with EDTA serum treatment to avoid the prozone effect, or titration, may be very helpful to enhance robustness of the assay. Finally, in cases where MFI values are neither “low” nor “high” (within the range of 2,000–10,000), complement activating potential or IgG subtypes of DSA (21) or intragraft DSA (18) assays may help to stratify risk of graft loss, and as such should be further explored.

The retrospective nature of our study is a limitation, although it did allow for the identification of the peak time point with sufficient followup. The peak time point was chosen for AMR^Neg^ patients as the sample with the highest sum MFI value to allow for maximal alloantibody assault, and thereby the most appropriate to compare against AMR^Pos^ MFI values. This retrospective analysis will have to be prospectively validated, ideally in a multicentric study allowed by the recent ISHLT consensus for AMR diagnosis in LTx (12). Importantly, given the differences between the two single antigen test suppliers, our results only apply for the One Lambda^®^ platform. Therefore, specific validation of this analysis should be also performed using the Immucor^®^ single antigen platform. Moreover, according to a 20% coefficient of variation allowance (22), MFI quantification deemed to be driven by antigen density at the surface of the bead, antibody:antigen affinity, and the prozone effect, should be interpreted using flexible thresholds. Lastly, current SAB tests only explore DSA directed against HLA, and a high-throughput detection tool for non-HLA DSA is not yet available to guide clinical care. This type of test would be particularly relevant in the case of an AMR clinical pattern with low MFI HLA DSA or without DSA (23).

## Conclusion

We demonstrate that AMR^Pos^ LT patients have higher number of specificities and increased MFI values when compared to AMR^Neg^ patients. DQ DSAs were associated with poorer clinical outcomes through AMR occurrence.

Even so, given the wide distribution of MFI in both AMR^Pos^ and AMR^Neg^ groups, DSA results should be interpreted with high regard for clinical graft failure status and additional DSA testing such as IgG subtype, complement binding capacity or intragraft DSA detection should be evaluated for a more accurate risk assessment for graft loss.

## The Foch Lung Transplantation Group

**Dr Clément Picard, Dr. Dominique Grenet, Dr. Sandra De Miranda, Dr. Abdul Monem Hamid, Dr. Benoit Douvry, Dr. Laurence Beaumont-Azuar, Dr. Geneviève Le Bourdelles, Dr. Hélène Neveu, Dr. Daniela Usturoi, Dr. Marc Stern, Dr. Antoine Roux;** Pneumology Department, Foch Hospital, Suresnes France; **Dr. Alain Chapelier, Dr. Edouard Sage, Dr. Philippe Puyo, Dr. Pierre Bonnette, Dr. Jocelyn Bellier, Dr. Mathieu Glorion, Dr. Salam Abou-Taam, Dr. François Gonin, Dr. Triet Ngo**, Thoracic Surgery Department, Foch Hospital, Suresnes France; **Dr. Elise Cuquemelle, Dr. François Parquin**, Thoracic Intensive Care Unit, Foch Hospital, Suresnes France; **Dr Charles Cerf, Dr. Grégoire Trebbia, Dr. Alexis Soummer, Dr. Jêrome Devaquet, Dr. Anthony Lanceleur, Dr. Koceila Bouferrache, Dr. David Courtier, Dr. Vincent Caille, Dr. Anne-Gaëlle Si Larbi**, Intensive Care Unit, Foch Hospital, Suresnes France; **Dr. Marc Fischler, Dr. Morgan Le Guen, Dr. Virginie Dumans-Nizard, Dr. Barbara Szekely, Dr. Mireille Michel-Cherqui, Dr. Jean- Yves Marandon, Dr. Ngai Liu, Dr. Léa Ley, Dr. Julie Bresson, Dr. Marie Louise Felten, Dr. Béatrice Angemont, Dr. Valentina Assenzo, Dr. Olivier Belze, Dr. Marie Binczak, Dr. Patrick Clapson, Dr. Camille Cornet, Dr. Jean-Louis Dumoulin, Dr. Sébastien Jacqmin, Dr. Olivier Pruszkowski, Dr. Adrian Radu, Dr. Céline Roussel, Dr. Benoît Vandenbunder, Dr. Nicolas Verroust, Dr. Thibaut Mariaux de Serres**, Anesthesiology Department, Foch Hospital, Suresnes France; **Dr. Leila Zemoura; Dr. Yves Denoux; Sonia Holifanjaniaina; Dr. Elisabeth Longchampt; Dr. Jean-Marc Guinebretiére**, Pathology Department, Foch Hospital, Suresnes France; **Dr François Mellot; Dr. Axel Guth; Dr. Nadia Canepa**, Radiology Department, Foch Hospital, Suresnes France; **Dr. Marc Vasse, Dr. Eric Farfour, Dr. Emilie Cardot, Dr. Pierre Cahen, Dr. Philippe Lesprit, Dr. Damien Mathonnet**, Biology Department, Foch Hospital, Suresnes France; **Dr Sophie Hillaire, Dr. Christine Veyrie, Dr. Florence Bouilloud, Dr. Barbara Néraud**, Internal Medecine, Foch Hospital, Suresnes France; **Dr. Dominique Dardelle, Dr. Eve Camps**, Pharmacy Department, Foch Hospital, Suresnes France.

## Ethics Statement

This observational study was approved by the research protocol evaluation committee of the Institutional Review Board of the French Learned Society for Respiratory Medicine—Société de Pneumologie de Langue Française. Every patient in this study was enrolled in a prospective non-therapeutic interventional research protocol [either COLT (NCT00980967, ID-RCB:2009-A00036-51) or RhumTP (NCT01791166, ID-RCB:2010-A01174-35)]. Upon enrollment, all patients signed consent for research use of their clinical data.

## Author Contributions

Research design: AR, MS, FP, CS, and KT. Writing of the paper: AR, MS, FP, CS, and KT. Performance of the research: AR, MS, FP, CS, CG, IL, SH, CP, DG, SM, BD, LB-A, ES, JD, EC, MG, and AH. Analytic tools: MR and KT. Data analysis: AR, MS, FP, CS, KT, and IL.

## Conflict of Interest Statement

AR has conflicts of interest to disclose as described by the Transplant International journal: he served as a consultant for Novartis France (concerning CMV in solid organ transplantation). The other authors have no conflicts of interest to disclose.

## Abbreviations

Ab, antibody; AMR, antibody-mediated rejection; CLAD, chronic lung allograft dysfunction; DSA, donor-specific antibody; DQ DSA, anti-HLA DQ donor-specific antibody; Non DQ DSA, DSA without reactivity against HLA DQ antigen; FEV1, forced expiratory volume in 1 second; HT, heart transplantation; KT, kidney transplantation; LT, lung transplantation; MFI, mean fluorescence intensity; SAB, single antigen beads.
